# Radiation-induced sarcoma after radiotherapy for breast cancer: A retrospective case series

**DOI:** 10.1016/j.jpra.2025.02.017

**Published:** 2025-02-26

**Authors:** Samar Saad A Alshehri, Nader Ashraf, Ahmed Abdellatif, Abderrahman Ouban, Nuha A. Khoumais

**Affiliations:** a Breast Imaging Section, Department of Radiology, King Faisal Specialist Hospital and Research Center, Riyadh, Saudi Arabia; b College of Medicine, Alfaisal University, Riyadh, Saudi Arabia; c Department of Pathology, Alfaisal University, Riyadh, Saudi Arabia

**Keywords:** Radiation-induced, Radiotherapy, Breast cancer, Sarcoma, Angiosarcoma

## Abstract

Radiation-induced breast sarcoma (RIBS) is a rare yet serious complication of radiotherapy for breast cancer, with limited data from the Middle East. This case series examines four patients with RIBS at a tertiary healthcare center in Saudi Arabia, highlighting a notably short latency period and an uncommon histological pattern. The mean age at breast cancer diagnosis was 44.75 years, and RIBS developed at a mean age of 48.25 years after an average latency of 43 months. One patient with a TP53 mutation had the shortest latency (28 months), suggesting a possible genetic predisposition. Histopathological evaluation confirmed undifferentiated pleomorphic sarcoma in all cases, distinct from the more commonly reported angiosarcoma. Radiologic assessments, including mammography, ultrasound, PET-CT, and MRI, revealed diverse tumor locations, with no cases of lymphadenopathy at presentation. Management varied, with three patients undergoing surgical excision and two receiving chemotherapy, while one patient with metastatic disease was ineligible for surgery. Follow-up over a mean duration of 34.7 months demonstrated variable outcomes, including recurrence and progression. These findings emphasize the need for heightened awareness of RIBS, particularly in genetically predisposed patients, and highlight the importance of long-term surveillance in breast cancer survivors. The predominance of undifferentiated pleomorphic sarcoma raises questions regarding genetic and environmental factors in RIBS development. Further research is necessary to explore risk stratification, optimize treatment strategies, and improve outcomes for this rare but aggressive malignancy.

## Introduction

Breast sarcomas are rare, histologically heterogeneous, nonepithelial malignancies that arise from the mesenchymal breast tissue. Although radiotherapy is essential for breast cancer management, its long-term risks include secondary malignancies such as radiation-induced breast sarcomas (RIBS), which occur at an estimated rate of 0.3% over 15 years.^1^ Despite being rare, RIBS pose a serious challenge due to their aggressive nature, limited treatment options, and poor prognosis. The criteria for identifying malignancies as radiation-induced were established by Cahan et al. in 1948 and later refined by Arlen et al. in 1971.^2^^,^^3^ These include: [1] different histological features between the primary tumor and the present sarcoma, [2] the development of sarcoma in a previously irradiated field; [3] a latent period, and [4] histological confirmation of the sarcoma. While a latency period of 5–10 years is typically expected, shorter intervals have been reported, particularly in genetically predisposed patients.^4^^,^^5^ However, the minimum required interval remains debated.

RIBS encompasses multiple histological subtypes, with angiosarcomas being the most prevalent, while other forms, such as undifferentiated pleomorphic sarcoma (UPS), remain uncommon.^6^ The incidence and survival rates of post-radiation sarcomas vary significantly, influenced by histology, latency period, and treatment approach.^7^ This case series describes an unexpectedly short latency period and an unusual predominance of UPS histology, findings that warrant further discussion.

This report addresses a gap in regional data by presenting a case series from Saudi Arabia, emphasizing the role of genetic predispositions, clinical outcomes, and the need for heightened awareness of atypical RIBS presentations. This report explores possible contributing factors, including genetic susceptibility, environmental influences, and evolving radiotherapy techniques.

## Case series

This single-center retrospective study analyzed consecutive patients diagnosed with RIBS at our institution between January 2013 and December 2022. Cases were identified through a systematic review of institutional oncology, pathology, and radiology databases.

Patients were included if they had a prior history of primary breast cancer, no prior evidence of primary sarcoma or metastatic disease at diagnosis, and had received adjuvant radiotherapy before developing a histologically confirmed sarcoma within the irradiated field. Exclusion criteria included sarcomas arising outside the irradiated region, incomplete medical records, or histopathology inconsistent with radiation-induced malignancies. Based on these criteria, four female patients were identified.

The mean age at primary breast cancer diagnosis was 44.75 years (29–61), while the mean age at sarcoma diagnosis was 48.25 years (31–66) (Table 1). The latency period, defined as the interval between the completion of radiotherapy and the diagnosis of RIBS, had a mean duration of 43 months (28–60). Notably, one patient with a confirmed TP53 mutation exhibited the shortest latency of 28 months, compared to the longest latency of 60 months for a patient without known genetic mutations. The average total radiation dose administered was 42.625 Gy (40–50).

**Table 1:** Baseline characteristics and treatment of patients at initial breast cancer diagnosis.

Patient ID	Age at breast cancer diagnosis (year)	History	Histopathology	Axillary lymph node involvement	Radiotherapy dose & fractionation
**1**	29	Known Li-Fraumeni syndrome	IDC G2, ER+/PR -, HER2 -	Yes (single node)	40 Gy x 15 fractions over 3 wk
**2**	32	BRCA mutation and breast cancer family history	IDC G3, ER-/PR -, HER2+	Yes	40.5 Gy x 14 fractions over 3 wk
**3**	57	Bilateral breast cancer and DCIS	DCIS intermediate grade with necrosis	No	50 Gy x 25 fractions over 5 wk
**4**	61	No significant history	IDC G3 triple negative	No	40 Gy x 14 fractions over 4 wk

IDC, invasive ductal carcinoma; DCIS, ductal carcinoma in situ; ER, estrogen receptor; PR, progesterone receptor; HER2, human epidermal growth factor receptor 2.

**Figure 1:**
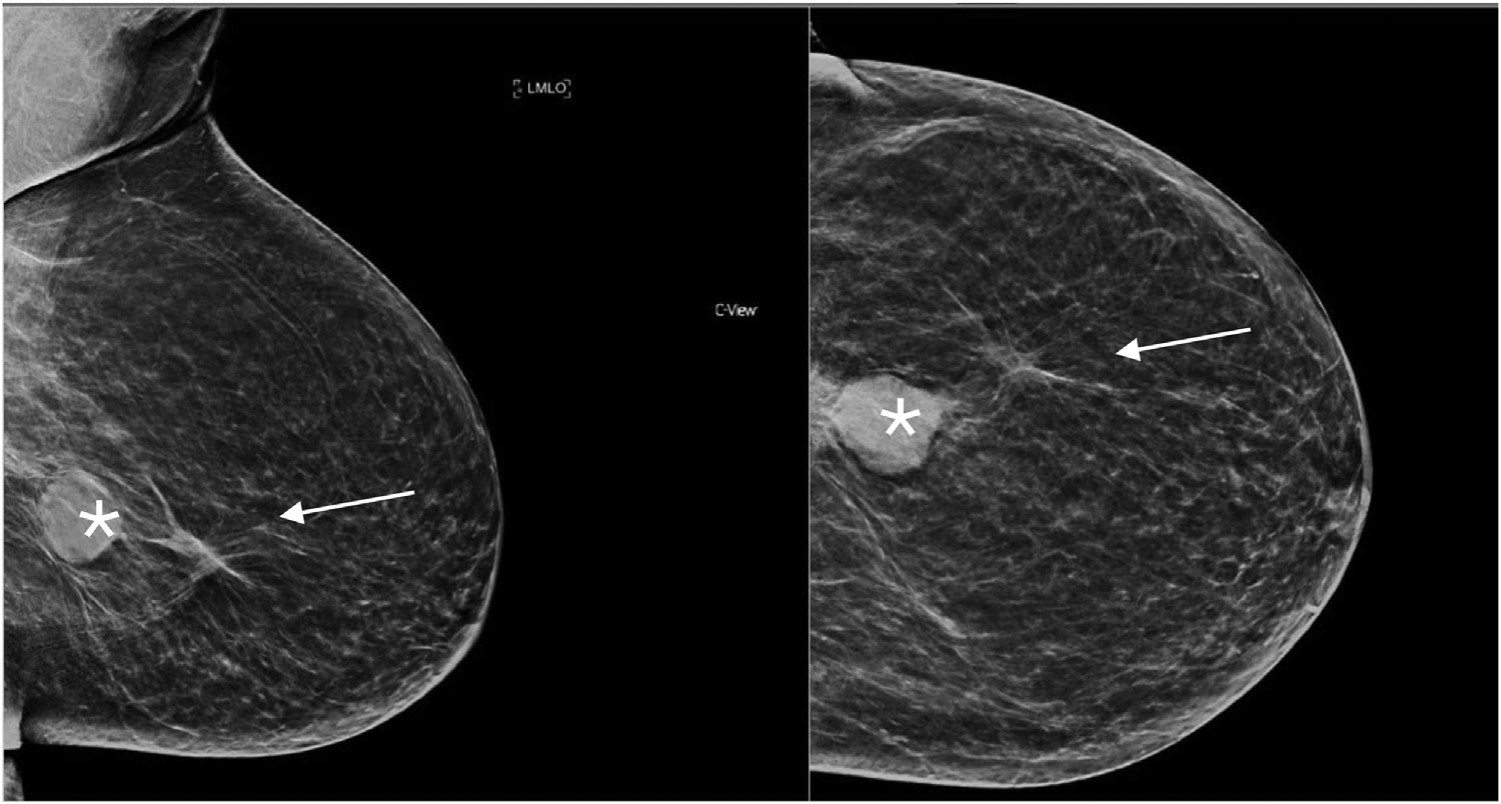
Mammogram shows a circumscribed high-density left lower central posterior third mass (*) with adjacent anterior post-operative asymmetry (white arrow), measuring about 2.4 × 2.7 × 3 cm.

**Figure 2:**
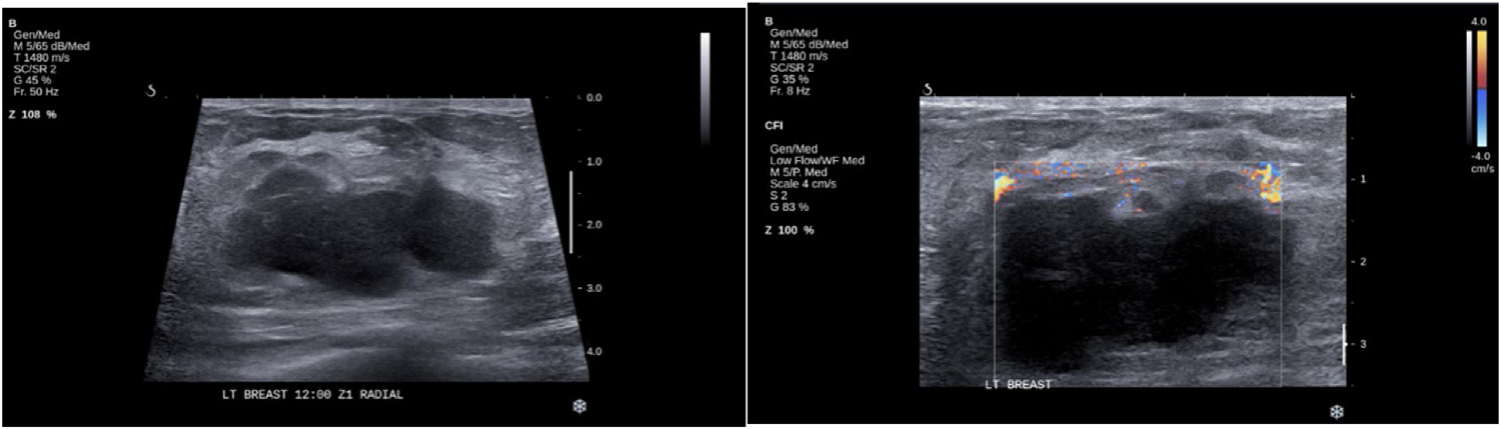
Ultrasound shows a heterogenous mainly hypoechoic irregular microlobulated solid large mass at 12 o'clock anterior third measures 4.9 × 3.2 × 4.8 cm with peripheral vascularity .

All four cases in this cohort were histologically classified as UPS (Table 2), an unusual finding given the predominance of angiosarcoma in RIBS literature. To validate the findings, all pathology samples were reviewed by an independent senior pathologist (AO). Radiological assessments, including mammography (Figure 1), ultrasound (Figure 2), PET-CT, and MRI (eFigure 1 and 2 in Supplementary Material), confirmed tumor locations and characteristics.

**Table 2:** Clinical characteristics, treatment, and outcomes of patients with radiation-induced breast sarcoma.

Patient ID	Age at sarcoma diagnosis (year)	Tumor location	Latency period (months)	Histopathology	Treatment	Disease course
**1**	31	Left axillary tail	28	UPS	Wide surgical excision followed by chemotherapy	No recurrence at 18-month follow-up
**2**	36	Left TRAM	48	UPS	Chemotherapy (Gemcitabine/ Docetaxel)	Progressive metastatic disease at 9-month follow-up
**3**	60	Left breast	36	UPS	Surgical excision only (patient refused chemotherapy)	Local recurrence at 12 months, subsequent progression
**4**	66	Left mastectomy bed	60	UPS	Surgical excision only (no chemotherapy due to multiple comorbidities)	Recurrence after 7 years, death from unrelated cause

UPS, undifferentiated pleomorphic sarcoma; TRAM, transverse rectus abdominis myocutaneous flap.

Three patients underwent surgical excision, while one was ineligible due to distant pulmonary metastases (Table 2). Two patients received chemotherapy; one declined adjuvant treatment, and another was deemed ineligible due to multiple comorbidities. Follow-up outcomes varied: one patient remained disease-free at last follow-up, another experienced recurrence within a year, and a third had no recurrence for seven years before dying from unrelated causes (Table 2). The patient with metastases exhibited disease progression over nine months.

## Discussion

Pleomorphic sarcoma is a rare breast sarcoma subtype, and secondary malignancy risks rise as breast cancer survival increases. While prior studies report RIBS latency exceeding five years, our series describes a mean latency of 43 months, with one case occurring as early as 28 months. Additionally, all four cases in our cohort were UPS, an uncommon histology for RIBS. These findings raise considerations regarding genetic predisposition, early tumorigenesis, and potential environmental or treatment-related influences on sarcoma development.

The latency period observed in our study is notably shorter than the 7.5–8.7 years reported in large retrospective cohorts by Yap et al. and Kirova et al.^1^^,^^6^ Prior research suggests genetic alterations, particularly TP53 (Li-Fraumeni syndrome) and BRCA mutations, may accelerate radiation-induced tumorigenesis . One patient in our study with a TP53 mutation developed RIBS in 28 months, reinforcing a possible genetic predisposition to early-onset post-radiation malignancies.^4^^,^^5^ Younger age at breast cancer diagnosis may also play a role, as some studies suggest shorter latency periods in younger patients, possibly due to variations in cellular proliferation and DNA repair mechanisms.^4^, ^5^, ^6^

The predominance of UPS in all four cases is another unexpected finding.^7^^,^^8^ Several factors could explain this observation. First, genetic predisposition may influence sarcoma histology, particularly in patients with TP53 mutations. Second, institutional referral and selection bias may have contributed, as cases were derived from a single tertiary center. Third, differences in histopathologic classification across institutions could account for variability in reported subtypes. While angiosarcomas are aggressive with rapid progression, UPS may have distinct radiologic and clinical characteristics, influencing its detection and diagnosis.

Although rare, RIBS presents significant clinical challenges due to limited treatment options and poor prognosis.^9^ The role of chemotherapy in RIBS remains controversial, with inconsistent evidence regarding its efficacy.^6^^,^^9^^,^^10^ The findings in our study highlight the need for individualized treatment strategies, with surgery remaining the mainstay for localized disease and chemotherapy considered on a case-by-case basis.

These findings have important implications for breast cancer survivors receiving radiotherapy. They underscore the necessity of long-term surveillance, particularly in genetically predisposed patients, where shorter latency periods could lead to earlier-onset RIBS. The predominance of UPS in our cohort suggests a broader histologic spectrum of post-radiation sarcomas than previously recognized. Given the limited regional data on RIBS, our study contributes to expanding the understanding of post-radiation sarcoma presentations in Middle Eastern populations and emphasizes the need for larger multi-center studies to validate these findings.

While valuable, this study has limitations. As a single-center retrospective series with a small sample size, generalizability is limited. The clustering of UPS cases may reflect selection bias rather than an epidemiologic trend. Future research should focus on prospective studies with genetic profiling to explore the interplay between radiotherapy, genetic susceptibility, and sarcoma histology.

In conclusion, our study presents an important case series of RIBS with shorter-than-expected latency periods and an unexpected predominance of UPS histology. Although these findings differ from previous reports, they highlight critical aspects of early tumorigenesis, genetic predisposition, and the need for long-term follow-up in breast cancer survivors who have undergone radiotherapy.

## Patient consent

Written, informed consent was obtained from the patients. They have reviewed and agreed to the details presented in this article, including the use of any accompanying images or data.

## Ethics approval

Not applicable.

## Author contributions

Samar Alshehri, Nader Ashraf, Ahmed Abdellatif, Abdurrahman Ouban, and Nuha Khoumais drafted the manuscript. Samar Alshehri and Nader Ashraf reviewed and finalized the manuscript. All authors have reviewed this manuscript and approved it for submission.

## Funding

This study did not receive any funding.

## Competing interests

The authors declare no conflict of interest.

## Appendix Supplementary materials

Image, application 1

## Footnotes

Supplementary material associated with this article can be found, in the online version, at doi:10.1016/j.jpra.2025.02.017.

## References

[1] Yap J., Chuba P.J., Thomas R. (2002). Sarcoma as a second malignancy after treatment for breast cancer. Int J Radiat Oncol*Biol*Phys..

[2] Cahan W.G., Woodard H.Q., Higinbotham N.L., Stewart F.W., Coley B.L. (1998). Sarcoma arising in irradiated bone. Cancer..

[3] Arlen M., Higinbotham N.L., Huvos A.G., Marcove R.C., Miller T., Shah I.C. (1971). Radiation-induced sarcoma of bone. Cancer.

[4] Mark R.J., Poen J., Tran L.M., Fu Y.S., Selch M.T., Parker R.G. (1994). Postirradiation sarcomas. A single-institution study and review of the literature. Cancer.

[5] Cha C., Antonescu C.R., Quan M.L., Maru S., Brennan M.F. (2004). Long-term results with resection of radiation-induced soft tissue sarcomas. Ann Surg.

[6] Kirova Y.M., Vilcoq J.R., Asselain B., Sastre-Garau X., Fourquet A. (2005). Radiation-induced sarcomas after radiotherapy for breast carcinoma: a large-scale single-institution review. Cancer.

[7] Bansal A., Kaur M., Dalal V. (2017). Pleomorphic sarcoma of breast: a report of two cases and review of literature. Acta Med Iran.

[8] Mery C.M., George S., Bertagnolli M.M., Raut C.P. (2009). Secondary sarcomas after radiotherapy for breast cancer: sustained risk and poor survival. Cancer.

[9] Brady M.S., Garfein C.F., Petrek J.A., Brennan M.F. (1994). Post-treatment sarcoma in breast cancer patients. Ann Surg Oncol.

[10] Barrow B.J., Janjan N.A., Gutman H. (1999). Role of radiotherapy in sarcoma of the breast–a retrospective review of the M.D. Anderson experience. Radiother Oncol.

