# Supplementary material for: Radiation-induced sarcoma after radiotherapy for breast cancer: A retrospective case series
Source: JPRAS Open. 2025 Feb 26;44:123–8. doi: 10.1016/j.jpra.2025.02.017 (PMC11976226; doi:10.1016/j.jpra.2025.02.017)
Supplement: Supplementary file 1 [file mmc1.pdf]

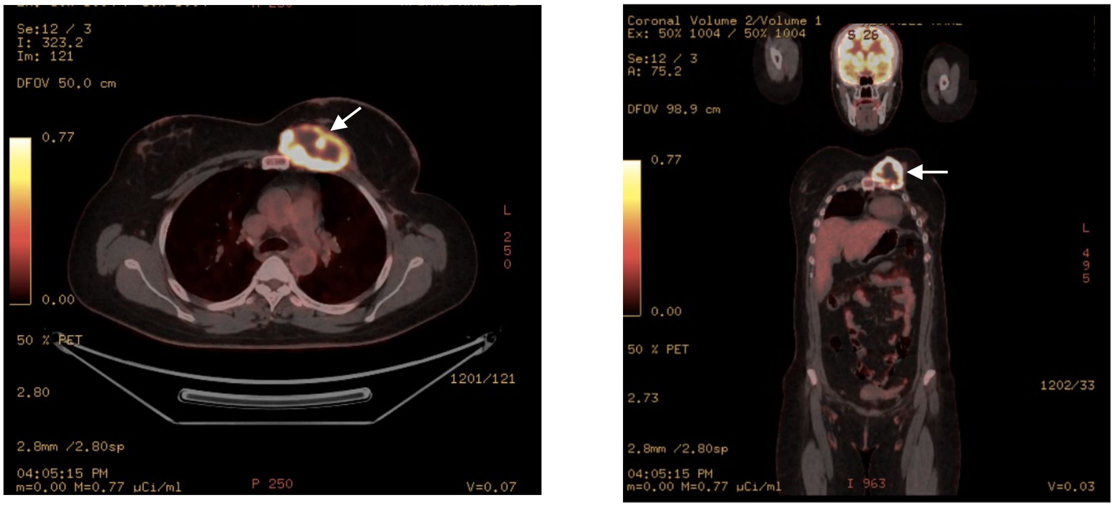

eFigure 1: PET-CT shows a hypermetabolic mass (white arrow) involving the medial aspect of the left chest wall with central necrosis, specifically, within the left subpectoral location measuring approximately 5.5 x 6.6 x 6 cm in its anteroposterior transverse and craniocaudal diameters.

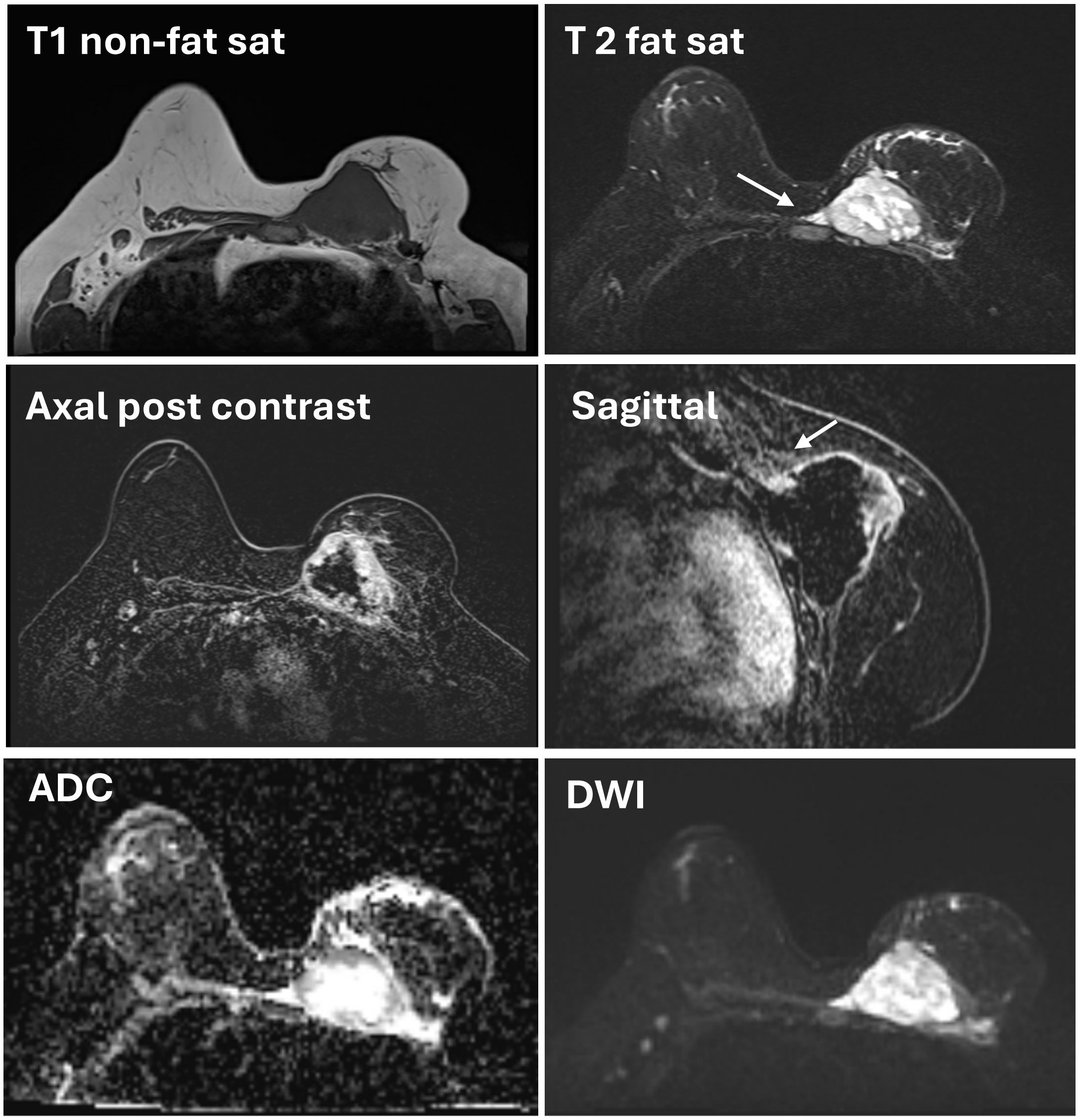

eFigure 2: MRI shows an irregular 6 x 5.7 cm mass noted in the upper inner quadrant of the reconstructed breast with necrotic center and peripheral irregular nodular enhancement which demonstrates diffusion restriction. The mass abuts the chest wall and is inseparable from the intercostal muscles, especially at the level of the second intercostal space. The pectoralis major muscle (white arrow) is thickened with edema and enhancement in keeping with invasion.
